# Quercetin modified electrospun PHBV fibrous scaffold enhances cartilage regeneration

**DOI:** 10.1007/s10856-021-06565-z

**Published:** 2021-08-10

**Authors:** Wei Chen, Yongsheng Li, Yuting Huang, Yao Dai, Tingfei Xi, Zheng Zhou, Hairong Liu

**Affiliations:** 1 College of Materials Science and Engineering, Hunan University, Changsha, 410082 China; 2 Shenzhen Institute, Peking University, Shenzhen, 518057 China; 3 College of Biology, Hunan University, Changsha, 410082 China; 4 Hunan Province Key Laboratory for Spray Deposition Technology and Application, Hunan University, Changsha, 410082 China

**Keywords:** Cartilage regeneration, PHBV, Surface modification, Quercetin, Tissue engineering

## Abstract

It suggests that the poly (3-hydroxybutyric acid-co-3-hydroxyvaleric acid) (PHBV) scaffold can be used for cartilage tissue engineering, but PHBV is short of bioactivity that is required for cartilage regeneration. To fabricate a bioactive cartilage tissue engineering scaffold that promotes cartilage regeneration, quercetin (QUE) modified PHBV (PHBV-g-QUE) fibrous scaffolds were prepared by a two-step surface modification method. The PHBV-g-QUE fibrous scaffold facilitates the growth of chondrocytes and maintains chondrocytic phenotype resulting from the upregulation of SOX9, COL II, and ACAN. The PHBV-g-QUE fibrous scaffold inhibited apoptosis of chondrocyte and reduced oxidative stress of chondrocytes by regulating the transcription of related genes. Following PHBV-g-QUE fibrous scaffolds and PHBV fibrous scaffolds with adhered chondrocytes were implanted into nude mice for 4 weeks, it demonstrated that PHBV-g-QUE fibrous scaffolds significantly promoted cartilage regeneration compared with the PHBV fibrous scaffolds. Hence, it suggests that the PHBV-g-QUE fibrous scaffold can be potentially applied in the clinical treatment of cartilage defects in the future.

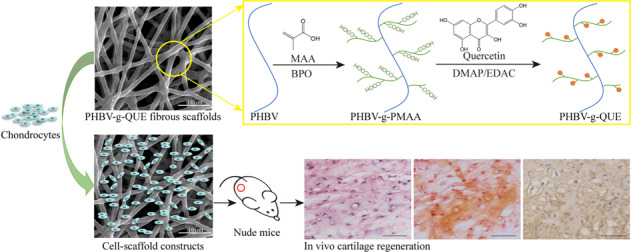

## Introduction

Articular cartilage is a very important tissue in the human body that acts as a shock absorber and reduces the friction of joints during movement [1]. Due to articular cartilage is neither innervated nor vascularized, cartilage damages caused by trauma, disease, and aging are difficult to repair by the cartilage itself [2, 3]. At present, surgical treatments of cartilage defects include osteochondral allograft or autologous transplant, microfracture, abrasion arthroplasty, etc. [4–6]. However, limited donor tissue reduces the application of osteochondral allograft, and the immune rejection of patients limits the practice of autologous transplant [6, 7]. Restoring cartilage defects via microfracture or abrasion arthroplasty only provides a short-term functional improvement due to cartilage defects filled with fibrocartilage rather than hyaline articular cartilage [8, 9]. Thus, the restoration of cartilage defect remains a major clinical challenge.

Cartilage tissue engineering (CTE), as a promising technique for repairing cartilage defects, can fill cartilage defects with hyaline articular cartilage that integrates with original articular cartilage [6]. It has been suggested that the proliferation and the chondrocytic phenotype of chondrocytes significantly influence the cartilage regeneration at the site of defects [10–12]. The regeneration of cartilage is benefited from chondrocytes maintaining chondrocytic phenotype, since the higher expression of cartilage extracellular matrix (ECM) related genes and lower transcription of metalloproteinases related genes have been discovered in those cells [13, 14]. The CTE scaffold plays a crucial role in cartilage regeneration, since it provides a favorable microenvironment for cell adhesion, proliferation and differentiation, and functional CTE scaffolds induce transcription of genes that facilitates chondrocytes maintaining their phenotype and forming new ECM of cartilage [15–17]. Therefore, it is the key for cartilage repair to manufacturing a functional CTE scaffold that enhances the efficiency of cartilage regeneration.

Poly (3-hydroxybutyric acid-co-3-hydroxyvaleric acid) (PHBV) is a suitable biomaterial for producing tissue engineering scaffolds due to its biocompatibility, biodegradability, non-toxicity and high mechanical properties [18–20]. However, PHBV lacks bioactivity that has limited its usage in tissue engineering applications [21, 22]. It has been reported that graft proteins or active factors onto the surface of PHBV can improve its biological activity, and the modified functional materials can regulate cell adhesion, proliferation, etc. [22–25]. Quercetin (3,3′,4′,5,7‐pentahydroxyflavone, QUE), a bioactive phytomolecule, has various biological and pharmacological characteristics, including anti-apoptotic, antioxidant, anti-inflammatory, anti-angiogenic and anticancer properties, etc. [26–29]. Importantly, it has been reported that QUE loaded hydrogel maintains the chondrocytic phenotype, inhibits ECM degradation, and delays the progression of OA [30, 31]. Hence, it may be a feasible way to improve the bioactivity of PHBV by surface modification with QUE that may significantly promote the regeneration of cartilage.

In this study, QUE modified PHBV (PHBV-g-QUE) fibrous scaffolds were prepared by a two-step surface modification method. Our data demonstrate that PHBV-g-QUE fibrous scaffolds exhibit multiple bioactivities that contribute to the regeneration of cartilage, including maintain the chondrocytic phenotype, promote the proliferation of chondrocytes, inhibit the apoptosis of chondrocytes, and release oxidative stress of chondrocytes. And PHBV-g-QUE fibrous scaffolds significantly enhance the efficiency of cartilage regeneration compared with PHBV fibrous scaffolds in vivo.

## Materials and methods

### Preparation of PHBV fibrous scaffolds and PHBV-g-QUE fibrous scaffolds

#### Preparation of electrospun PHBV fibrous scaffolds

PHBV (Mw = 300 kDa) with 3% HV was purchased from Tianan Biologic (China), and PHBV fibrous scaffolds were fabricated via electrospinning method. First, 1 g PHBV powders were dissolved in 20 mL liquid mixture containing dichloromethane and N, N-Dimethylformamide (DMF) (v/v = 9:1), by sonicating at 60 °C for 10 minutes to obtain a 5% (w/v) PHBV solution. Following the PHBV solution was transferred to a glass syringe with a steel needle, a voltage of 15 kV was applied to the needle tip. And then, the PHBV solution was pushed out at a flow rate of 5 mL/h. The prepared PHBV fibrous scaffold was formed and collected at the grounded collection plate, which is 15 cm away from the needle tip. All prepared PHBV fibrous scaffolds were dried under a fume hood for 48 h to volatilize the residual solvent.

#### Preparation of PHBV-g-QUE fibrous scaffold

The PHBV fibrous scaffolds were cleaned with 50% aqueous alcohol for 1 h and washed twice with double-distilled water (ddH_2_O). The reaction solution was prepared by dissolving 0.5 g benzoyl peroxide (BPO) and 5 mL methacrylic acid (MAA) in 100 mL 50% aqueous alcohol solution at 80 °C. Then the PHBV fibrous scaffolds were modified in this reaction solution for 105 min to get the polymethacrylic acid (PMAA) grafted PHBV (PHBV-g-PMAA) fibrous scaffolds. Every prepared PHBV-g-PMAA fibrous scaffold was washed with alcohol and ddH_2_O five times to remove unreacted MAA and free PMAA. Finally, in the modified phosphate buffer saline (PBS) solution containing 2 mg/mL QUE, 5 mg/mL N-(3-Dimethylaminopropyl)-N-ethylcarbodiimide hydrochloride (EDAC) and 2.5 mg/mL 4-dimethylaminopyridine (DMAP) the PHBV-g-PMAA fibrous scaffolds reacted with QUE for 24 h at room temperature to form PHBV-g-QUE fibrous scaffolds via the esterification reaction. The prepared PHBV-g-QUE fibrous scaffolds were cleaned with ddH_2_O five times and lyophilized for 24 h for further use.

### Characterization of PHBV fibrous scaffolds and PHBV-g-QUE fibrous scaffolds

In order to explore the differences between the unmodified and modified PHBV fibrous scaffolds, the toluidine blue O (TBO) staining method was used to measure the surface carboxyl density of samples according to the previously established method [32], and attenuated total reflectance Fourier transform infrared spectroscopy (ATR-FTIR) (Thermo, American) was used to investigate the surface chemical structure of tested scaffolds. Moreover, the morphology of PHBV fibrous scaffolds and PHBV-g-QUE fibrous scaffolds was observed by scanning electron microscope (SEM) (FEI Quanta 200, FEI Company, USA), and the diameter of scaffolds was calculated by the Image J software.

### Cell culture with PHBV fibrous scaffolds and PHBV-g-QUE fibrous scaffolds

#### Rabbit chondrocytes isolation and culture

All animal experiments in this study were approved by the Animal Care and Ethics Committee of Hunan Academy of Chinese Medicine. Rabbit articular chondrocytes were isolated and cultured according to our previous publication [33]. In brief, articular cartilage was isolated from the knee joints of New Zealand white rabbits (1 week old) and cut into small pieces (<1 mm^3^). The cartilage pieces were transferred into centrifugal tubes and added adequate amounts of high glucose DMEM medium (HyClone, USA) containing 15% (w/v) type II collagenase (300 U/mg, Worthington, USA) to digest the ECM. Chondrocytes were collected after 4 h and 8 h digestion, respectively. The collected chondrocytes were cultured in cell culture dishes with high glucose DMEM medium containing 10% fetal bovine serum (Gibco, USA). The culture medium was changed every 2 days and chondrocytes at passage 2 were used for this study.

#### Cellular proliferation and morphology

In total, 5 × 10^3^ chondrocytes were seeded onto every tested sample located in a 24-well plate respectively. The proliferation rate of chondrocytes was determined by the Alamar Blue method following 1, 3, 5, and 7 days incubation respectively according to the protocol described in our previous publication [34]. The morphology of chondrocytes was detected by fluorescein diacetate (FDA) staining following 2, 4, 6, 8, and 10 days incubation respectively according to the established method in our previous publication [25].

#### RNA isolation and real-time quantitative polymerase chain reaction analysis

Total RNA was isolated from chondrocytes after 10 days for the real-time quantitative polymerase chain reaction (RT-qPCR) analysis. Firstly, total cellular RNA was isolated by lysing in Trizol (Invitrogen, USA) and the trace DNA contamination of samples was cleaned by DNase I (Fermentas, Canada). Then, stand complementary DNA (cDNA) synthesis reactions were performed by using the PrimeScript^TM^ Reverse Transcriptase (TaKaRa, Japan). RT-qPCR assay was performed using UltraSYBR Mixture (CWBIO, China) and the glyceraldehyde-3-phosphate dehydrogenase (*GAPDH*) gene was used as the internal control. The primers sequences of tested genes were designed by oligo software and validated by BLAST (Table 1).

**Table 1:** The sequence of primers used for RT-qPCR

Gene	5ʹ-3ʹ	Primer
*GAPDH*	Forward	TTGTCGCCATCAATGATCCAT
	Reverse	GATGACCAGCTTCCCGTTCTC
*SOX9*	Forward	GCGTCAACGGCTCCAGCAAGA
	Reverse	GCGTTGTGCAGGTGCGGGTAC
*ACAN*	Forward	GCTGCTACGGAGACAAGGATG
	Reverse	CGTTGCGTAAAAGACCTCACC
*COL* II	Forward	GAGAGCCTGGGACCCCTGGAA
	Reverse	CGCCTCCAGCCTTCTCGTCAA
*COL* I	Forward	CTAGCCACCTGCCAGTCTTTA
	Reverse	GGACCATCATCACCATCTCTG
*MMP1*	Forward	TTCCAAAGCAGAGAGGCAATG
	Reverse	CACCTGGGTTGCTTCATCATC
*MMP3*	Forward	GTGATACGCAAGCCCAGGTGT
	Reverse	CTCTTGGCAGATCCGGTGTGT
*MMP13*	Forward	GTCTTCTGGCTCACGCTTTTC
	Reverse	GGCAGCAACGAGAAACAAGTT
*APAF1*	Forward	TCGTGGTCTGCTGATGGTGCT
	Reverse	TGCTGTTACGGCCTGTTTGGA
*Bcl-2*	Forward	CGGAAGGGACTGGACCAGAGA
	Reverse	GCTGTCATGGGGATCACCTCC
*CASP3*	Forward	AAGCCACGGTGATGAAGGAGT
	Reverse	TCGGCAAGCCTGAATAATGAA
*Nrf2*	Forward	ATTCTTTCGGCAGCATCCTCT
	Reverse	CTGGGTTCAGCTATGAAGGCA
*SOD1*	Forward	GCACGGATTCCATGTCCACCA
	Reverse	TCACATTACCCAGGTCGCCCA
*SOD2*	Forward	CAGAAGCACAGCCTCCCCGAC
	Reverse	CCGTGGCGTTCAGGTTGTTCA

### Cartilage regeneration evaluation in vivo

The cartilage formation capacity of the PHBV fibrous scaffolds and PHBV-g-QUE fibrous scaffolds in vivo was tested by the subcutaneous implantation assay in nude mice. First, 4 × 10^5^ chondrocytes were seeded onto the PHBV fibrous scaffolds and PHBV-g-QUE fibrous scaffolds and cultured in vitro for 3 days. Then, specimens were folded and implanted subcutaneously into nude mice (6 weeks old, female).

After implantation for 4 weeks, the nude mice were sacrificed and the implanted specimens were collected. Specimens were fixed in 4% paraformaldehyde solution for 48 h. Fixed specimens were dehydrated with a graded series of 70, 80, 85, 90, 95, and 100% ethanol and vitrified by dimethylbenzene. Then specimens were embedded in paraffin and cut into 6 μm sections. Hematoxylin and Eosin (H&E) (Solarbio, China) and Safranin-O (SO) (Solarbio, China) staining were applied to detect the morphological and cartilage-specific ECM distribution of the specimens. To evaluate the type II collagen deposition of the specimens, immunohistochemical staining of the tissue sections was performed by using antibodies against type II collagen (Santa Cruz, USA, sc-52658). The H&E, SO and type II collagen staining were performed by following the standard laboratory protocol.

### Statistical analysis

All data in our study are expressed as means ± standard deviation for a minimum of *n* = 3. Differences between experimental groups were determined by the Student *t* test. The differences were considered at *p < 0.05 **p < 0.01 ***p < 0.001.

## Results

### Preparation and characterization of scaffolds

**Fig. 1:**
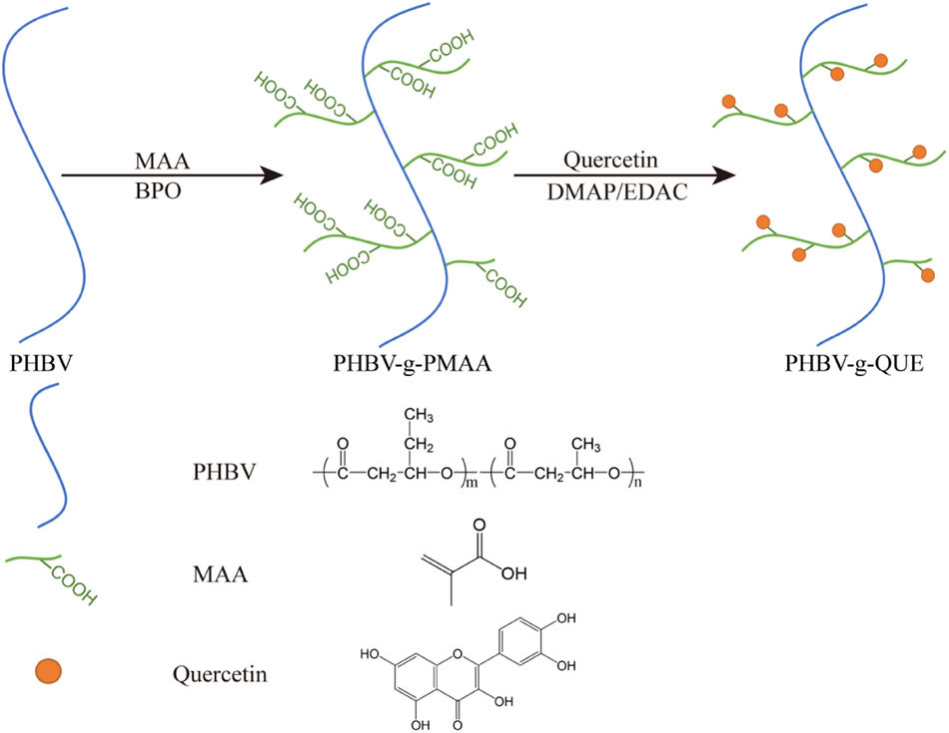
Schematic illustration of the QUE modification method

PHBV fibrous scaffolds were fabricated by the electrospinning method, and then PHBV-g-QUE fibrous scaffolds were prepared via two-step surface modification on PHBV fibrous scaffolds (Fig. 1). First, MAA was grafted to the surface of PHBV fibrous scaffolds, which introduced the carboxyl group onto their surface. Then, QUE was immobilized onto the surface of PHBV-g-PMAA fibrous scaffolds resulting from ester bond forming between hydroxyl groups of QUE and carboxyl groups of PMAA.

In order to investigate whether QUE was successfully immobilized on the surface of PHBV fibrous scaffolds, TBO staining and ATR-FTIR analysis were used to characterize the chemical functional groups of these prepared samples.

**Fig. 2:**
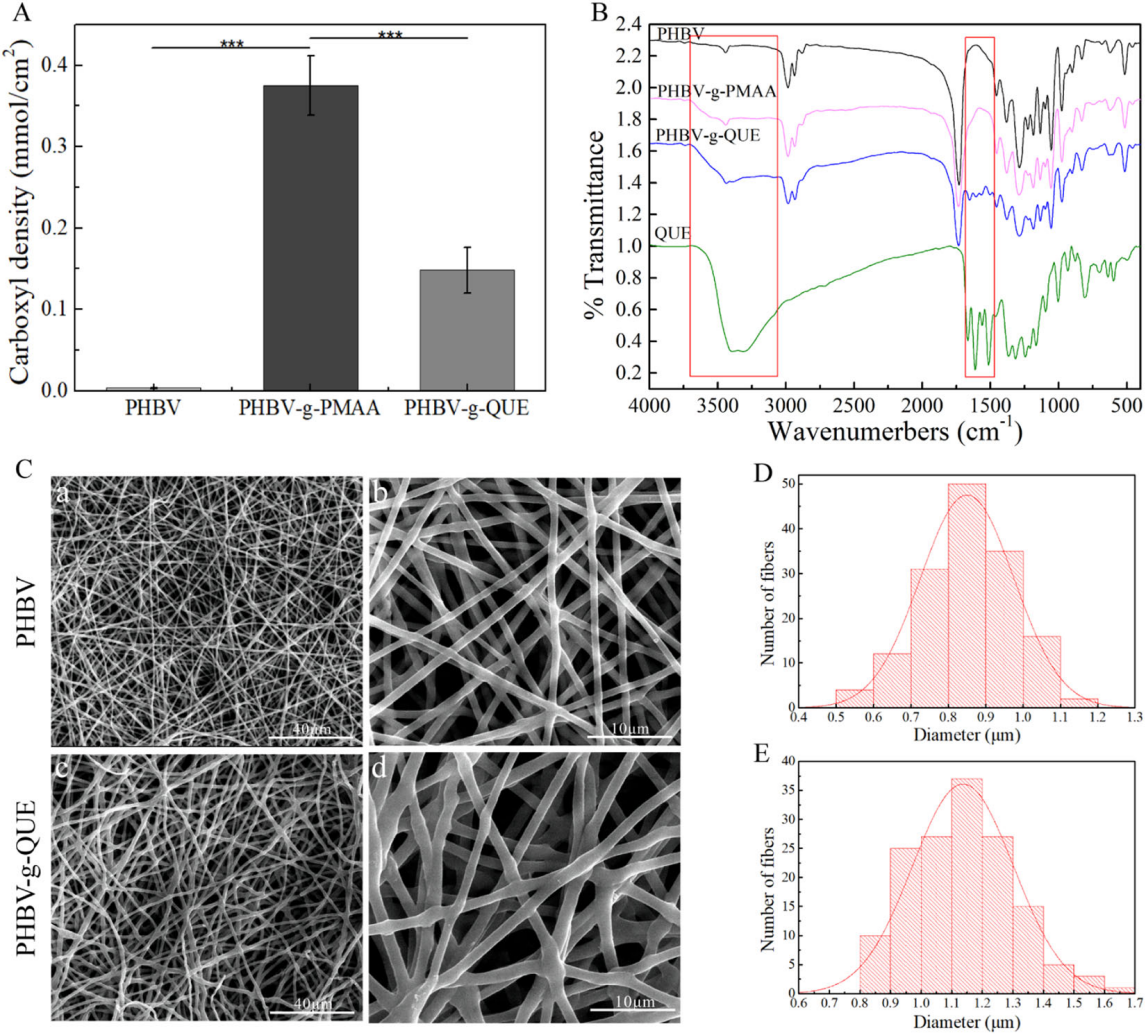
Characterization of scaffolds. (**A**) The surface carboxyl group density on scaffolds, (**B**) the FTIR spectrum of tested samples, (**C**) the microstructure of tested scaffolds, and the diameter of PHBV fibrous scaffold (**D**) and PHBV-g-QUE fibrous scaffolds (**E**)

As shown in Fig. 2A, the surface carboxyl group density on PHBV fibrous scaffolds was only 0.003 mM/cm^2^. But the surface carboxyl group density on PHBV-g-PMAA fibrous scaffolds significantly increased to 0.375 mM/cm^2^, demonstrating that the carboxyl group was introduced into the surface of PHBV fibrous scaffold successfully. The surface carboxyl group density on PHBV-g-QUE fibrous scaffolds decreased to 0.148 mM/cm^2^, indicating that the majority of carboxyl groups were consumed by the esterification reaction to immobilize QUE (Fig. 2A).

To further confirm QUE modification on the surface of prepared scaffolds, tested samples were examined by ATR-FTIR (Fig. 2B). The FTIR spectrum at 3100–3700 cm^−1^ is corresponding to the stretching vibration of -OH. Compared with PHBV fibrous scaffolds, the broad peak at 3100–3700 cm^−1^ was observed in the PHBV-g-PMAA fibrous scaffolds, indicating that PMAA was successfully grafted onto the surface of PHBV fibrous scaffolds. After QUE modification, the broad peak at 3100–3700 cm^−1^ was increased due to the abundance of -OH group in QUE molecules. In addition, it exhibited four additional absorbance bands at 1450–1650 cm^-1^, which represent the vibration of the skeleton structure of the QUE benzene ring. All these data demonstrate that PHBV-g-QUE fibrous scaffolds were successfully manufactured by the QUE modification method displayed in Fig. 1.

The microstructure of the PHBV fibrous scaffolds and PHBV-g-QUE fibrous scaffolds were characterized by SEM (Fig. 2C), and it demonstrated that the morphology of these two tested samples was similar. Fibers of the two samples were uniform and smooth, and no beading and surface defects were observed (Fig. 2C). The average diameter of PHBV fibrous scaffold was 0.85 ± 0.13 μm (Fig. 2D), and that of PHBV-g-QUE fibrous scaffolds was 1.13 ± 0.17 μm (Fig. 2E). These results indicated that the QUE modification did not significantly affect the morphology of PHBV fibrous scaffolds.

### PHBV-g-QUE fibrous scaffolds promote the proliferation of chondrocytes

**Fig. 3:**
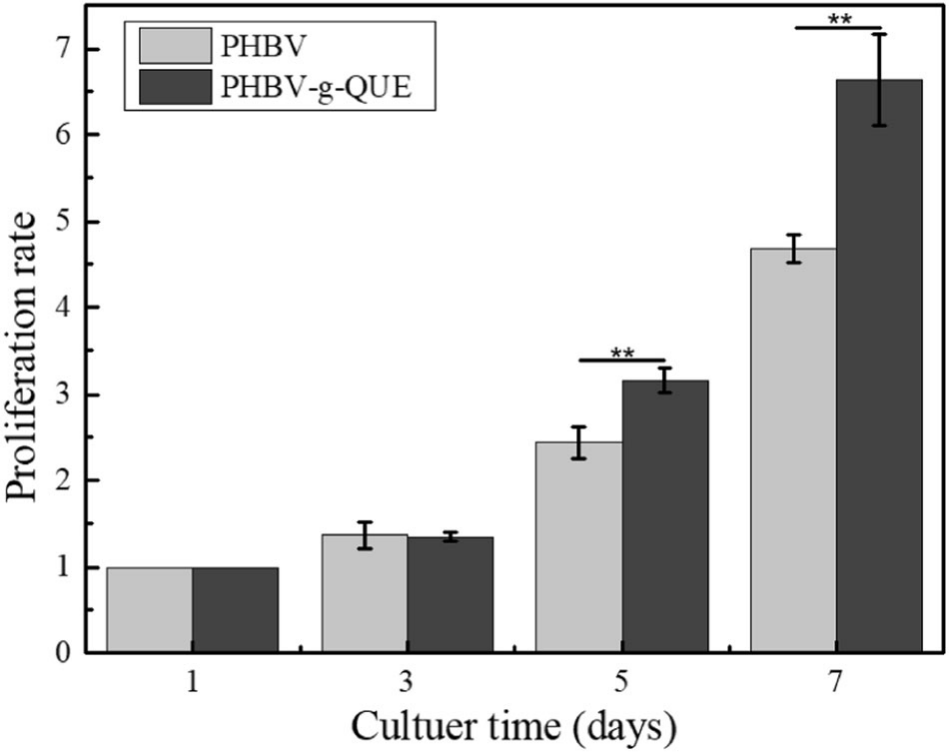
The proliferation rate of chondrocytes cultured on PHBV fibrous scaffolds and PHBV-g-QUE fibrous scaffolds

Since the proliferation of chondrocytes on the scaffold facilitates cartilage regeneration [35], whether PHBV-g-QUE fibrous scaffolds influence the growth of chondrocytes was tested by using the Alamar Blue assay. Following chondrocyte seeded on the PHBV fibrous scaffolds and the PHBV-g-QUE fibrous scaffolds, all samples were detected with Alamar Blue assay after 1, 3, 5, and 7 days incubation (Fig. 3). The proliferation rate of chondrocytes cultured with PHBV-g-QUE fibrous scaffolds was significantly higher than that cultured with PHBV fibrous scaffolds following 5 and 7 days incubation (*p* < 0.01). It suggested that PHBV-g-QUE fibrous scaffolds benefited the proliferation of chondrocytes compared with PHBV fibrous scaffolds.

### PHBV-g-QUE fibrous scaffolds maintain the chondrocytic phenotype

It suggests that the chondrocytic activity and phenotype of chondrocytes are crucial for cartilage regeneration [14]. Hence, the effect of PHBV-g-QUE fibrous scaffolds on chondrocyte phenotypic maintenance was tested by FDA staining and RT-qPCR analysis.

**Fig. 4:**
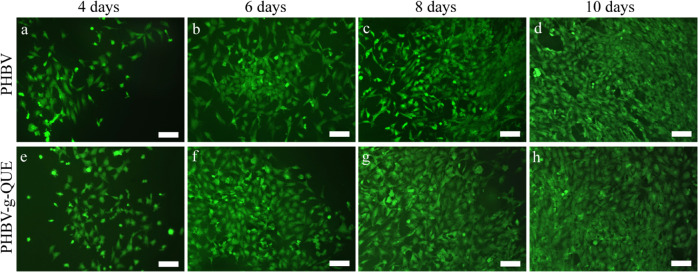
The morphology of chondrocytes cultured on PHBV fibrous scaffolds and PHBV-g-QUE fibrous scaffolds. Scale bar, 100 μm

The results of FDA staining showed that chondrocytes cultured with PHBV-g-QUE fibrous scaffolds and PHBV fibrous scaffolds all displayed typical chondrocyte morphology that appeared round or polygonal shapes (Fig. 4a, b, e, and f) during the 6 days incubation period. When the chondrocytes were incubated with tested samples for 8 and 10 days, chondrocytes cultured on PHBV fibrous scaffolds exhibited fibroblast-like shapes (Fig. 4c and d), but chondrocytes cultured on PHBV-g-QUE fibrous scaffolds remained round or polygonal morphology (Fig. 4g and h).

**Fig. 5:**
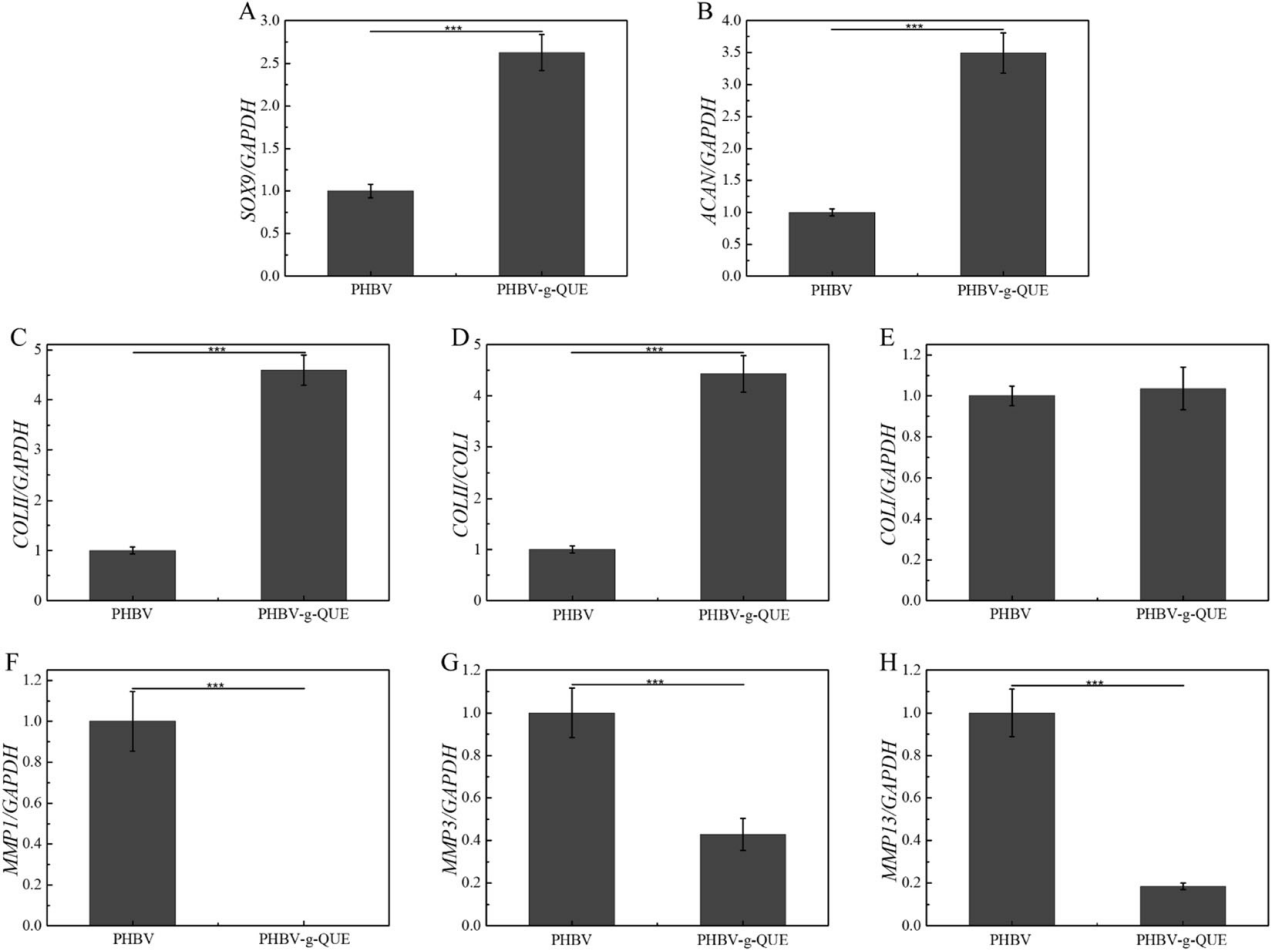
Transcription levels of cartilage matrix formation and degradation-related genes in chondrocytes cultured with PHBV fibrous scaffolds and PHBV-g-QUE fibrous scaffolds. (**A**) SOX9, (**B**) ACAN, (**C**) COLII, (**D**) the ratio of COLII/COLI, (**E**) COLI, (**F**) MMP1, (**G**) MMP3, (**H**) MMP13

Since the morphology alteration of chondrocyte may result from its dedifferentiation, the transcription of chondrocytic phenotype related genes in chondrocytes cultured with scaffolds were detected by using the RT-qPCR assays (Fig. 5). Compared with chondrocytes cultured with PHBV fibrous scaffolds, the transcription of SOX9, COLII and ACAN were significantly increased in ones cultured with PHBV-g-QUE fibrous scaffolds (Fig. 5A-C). MMP1, MMP3 and MMP13 are genes concerned with joint cartilage matrix degradation and were down-regulated in chondrocytes cultured with PHBV-g-QUE fibrous scaffolds compared with PHBV fibrous scaffolds (Fig. 5F-H). Moreover, the ratio of the mRNA level of COLII versus that of COLI (COLII/COLI), which represents the level of chondrocytic activity, in chondrocytes was also detected and calculated. It showed that the value of COLII/COLI was 4.43-fold higher in chondrocytes cultured with PHBV-g-QUE fibrous scaffolds than those cultured with PHBV fibrous scaffolds (Fig. 5D).

### Effects of PHBV-g-QUE fibrous scaffolds on chondrocyte apoptosis and oxidative stress

**Fig. 6:**
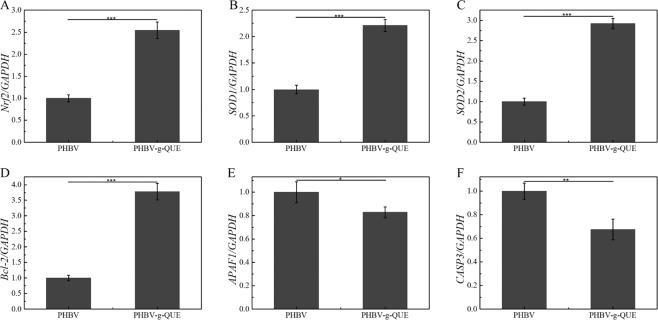
Transcription levels of oxidative stress-related genes and apoptosis-related genes in chondrocytes cultured with PHBV fibrous scaffolds and PHBV-g-QUE fibrous scaffolds. (**A**) Nrf2, (**B**) SOD1, (**C**) SOD2, (D) Bcl-2, (**E**) APAF1, (**F**) CASP3

Apoptosis of chondrocytes plays a primary role in the deterioration of cartilage diseases, and inhibiting chondrocyte apoptosis is very important for cartilage defect repair via the CTE approach [36, 37]. Oxidative stress is one of the causes of cell apoptosis [38]. Nrf2, SOD1, and SOD2 are proteins that reduce the oxidative stress of cells [39]. The result showed that the transcription levels of Nrf2 in chondrocytes cultured with PHBV-g-QUE fibrous scaffolds were significantly upregulated compared with those incubated with PHBV fibrous scaffolds (Fig. 6A). Compared with PHBV fibrous scaffolds, PHBV-g-QUE fibrous scaffolds significantly promoted the transcription of SOD1 and SOD2 in chondrocytes (Fig. 6B and C). The cellular apoptosis requires the participation of CASP3 and APAF1, and it is inhibited by Bcl-2, an apoptotic suppressor [40]. Compared with PHBV fibrous scaffolds, PHBV-g-QUE fibrous scaffolds significantly upregulated the transcription of Bcl-2 and downregulated the transcription of APAF1 and CASP3 in chondrocytes (Fig. 6D–F). It indicated that the PHBV-g-QUE fibrous scaffold could inhibit the apoptosis of chondrocytes and reduce the oxidative stress in chondrocytes.

### PHBV-g-QUE fibrous scaffolds enhance cartilage regeneration in vivo

To test whether PHBV-g-QUE fibrous scaffolds enhance cartilage regeneration in vivo, the cartilage regeneration efficiency of tested samples was examined following subcutaneous implantation in nude mice. Before implantation, chondrocytes were seeded onto the PHBV fibrous and PHBV-g-QUE scaffolds respectively and were incubated with scaffolds in vitro for 3 days. Then, all tested scaffolds with seeded chondrocytes were folded and implanted subcutaneously into nude mice for 4 weeks.

**Fig. 7:**
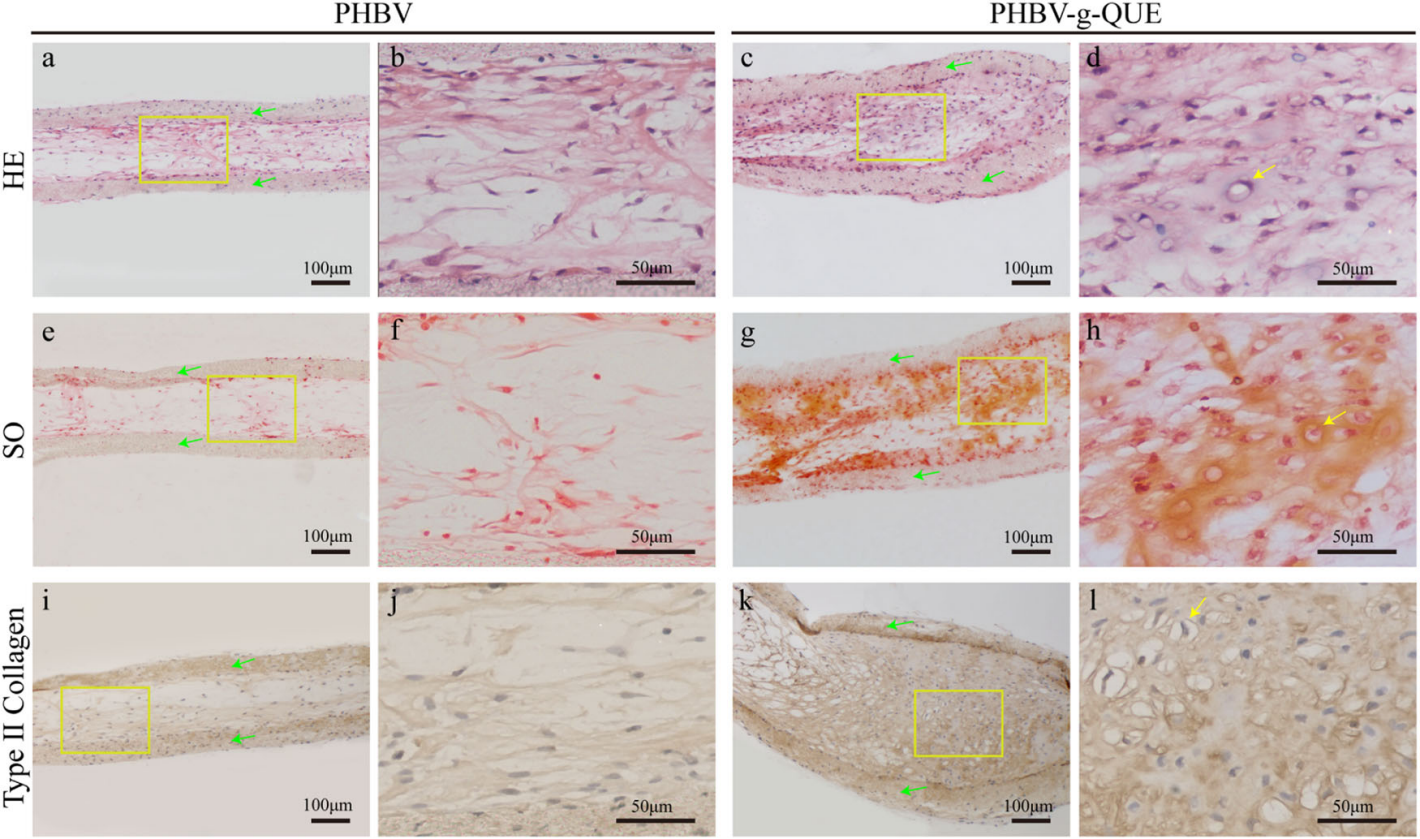
Histological analysis after the tested samples were implanted subcutaneously into nude mice for 4 weeks. (**a–d**) H&E staining, (**e-h**) SO staining, and (**i-l**) immunohistochemical staining of type II collagen. The green arrows indicate the scaffolds, and the yellow arrows indicate the cartilage lacuna

Within 4 weeks after subcutaneous transplantation, the living activities of nude mice were normal, and they did not appear any obvious inflammatory reaction as well. The morphological and cartilage-specific ECM distribution of specimens were detected by HE staining and SO staining respectively, and the type II collagen deposition of specimens was examined by immunohistochemical staining of type II collagen. Compared with the PHBV fibrous scaffold group (Fig. 7a and b), more cell aggregation and uniformed distribution were observed in the PHBV-g-QUE fibrous scaffold group (Fig. 7c and d). The SO and type II collagen staining intensity of the PHBV-g-QUE fibrous scaffold group (Fig. 7g, h, k and l) was significantly stronger than that of the PHBV fibrous scaffold group (Figs. 7e, f, i and j), indicating that PHBV-g-QUE fibrous scaffolds significantly promoted the formation of cartilage ECM. Compared with the PHBV fibrous scaffold, the PHBV-g-QUE fibrous scaffold enhanced the regeneration of cartilage-like tissue, since the area of cartilage-like tissue observed in the specimen of PHBV-g-QUE fibrous scaffold group (Fig. 7g and k) was significantly larger than that of PHBV fibrous scaffold group (Fig. 7e and i). In addition, mature cartilage lacunae were only observed in the PHBV-g-QUE fibrous scaffold group (Fig. 7d, h and i), but no cartilage lacunae were observed in the PHBV fibrous scaffold group (Fig. 7b, f, and j). It suggested that the PHBV-g-QUE fibrous scaffold could enhance cartilage regeneration in vivo by promoting the formation and maturation of neo-cartilage tissue.

## Discussion

Due to the extremely limited self-repair ability of the articular cartilage, the repair of cartilage defect is a major clinical challenge. CTE is a promising technique for repairing cartilage defects, while the slow regeneration of cartilage tissue after implantation and the relatively long recovery time limit its application in clinical treatments [41]. Since the scaffold plays a key role in tissue engineering, a functionalized scaffold that enhances cartilage regeneration could significantly reduce the time consumption of CTE [15, 16]. In this study, the PHBV fibrous scaffold was modified with QUE to fabricate a bioactive CTE scaffold that promotes cartilage regeneration.

It demonstrates that PHBV is a suitable biomaterial for producing CTE scaffolds due to its excellent biocompatibility, biodegradability and mechanical properties [20, 42], but the inert nature of the PHBV scaffold limits its application [21, 43]. Surface modification is an effective way to introduce bioactive substances into the surface of PHBV scaffolds [22, 25]. QUE, as a bioactive phytomolecule, exhibits multiple functions, which benefit chondrocytes keeping chondrocytic activity and growth that result in efficient cartilage regeneration [30, 31, 44]. QUE modified PHBV scaffold may display both advantages of PHBV and QUE that make it more applicable in CTE. To test this hypothesis, the PHBV-g-QUE fibrous scaffold was prepared and its influence on chondrocytes and the formation of cartilage tissue were tested in this study.

QUE was immobilized on the surface of the PHBV fibrous scaffold by a two-step surface modification method (Fig. 1). As a member of the flavonoid family, QUE has multiple hydroxyl groups that can be immobilized on the scaffold through the esterification reaction. The TBO staining and ATR-FTIR analysis result indicated that the QUE has been successfully grafted on the surface of PHBV fibrous scaffolds, and the SEM result demonstrated that the PHBV fibrous scaffolds and PHBV-g-QUE fibrous scaffolds showed similar fiber morphology (Fig. 2).

To test the bioactivity of PHBV-g-QUE fibrous scaffolds, its influence on chondrocytes was investigated. Compared with PHBV fibrous scaffolds, the PHBV-g-QUE fibrous scaffold could significantly promote the proliferation of chondrocytes (Figs. 3, 4). Our data suggested that the PHBV-g-QUE fibrous scaffold kept chondrocytic phenotype by upregulating the transcription of the cartilage matrix formation-related genes (including SOX9, COLII, and ACAN) and suppressing the transcription of cartilage matrix degradation-related genes (including MMP1, MMP3, and MMP13) of chondrocytes (Fig. 5). The results were consistent with the study on the encapsulation of QUE in hydrogel [30], suggesting that the chemical modification method did not abolish the functions of QUE. Moreover, the PHBV-g-QUE fibrous scaffold reduced the oxidative stress in chondrocytes and inhibited cell apoptosis by upregulating the transcription of Bcl-2, Nrf2, SOD1, and SOD2 and downregulating the transcription of APAF1 and CASP3 in chondrocytes (Fig. 6), which are similar to the anti-apoptotic and antioxidant of QUE [26, 27].

To confirm that the PHBV-g-QUE fibrous scaffold enhances cartilage regeneration in vivo, both PHBV-g-QUE fibrous scaffolds and PHBV fibrous scaffolds with adhered chondrocytes were implanted into nude mice for 4 weeks. Histological analysis results indicated that more cells and cartilage-specific ECM were observed from specimens of the PHBV-g-QUE fibrous scaffolds group (Fig. 7). In addition, the PHBV-g-QUE fibrous scaffold group showed typical cartilage features with abundant cartilage lacuna structures, but the PHBV fibrous scaffold group showed less cartilage-specific ECM secretions with immature chondrocytes (Fig. 7). It illustrates that the PHBV-g-QUE fibrous scaffolds enhance the formation and maturation of neo-cartilage tissue in vivo.

In summary, the PHBV-g-QUE fibrous scaffold significantly enhances cartilage regeneration, suggesting that it can be potentially applied for clinic treatment of cartilage defects in the future.

## Conclusion

In this study, electrospun PHBV fibrous scaffolds were modified with QUE to enhance the bioactivity of PHBV, which resulted in efficient cartilage regeneration. Prepared PHBV-g-QUE fibrous scaffolds promote the proliferation of chondrocytes, maintain the chondrocytic phenotype, and facilitate the formation of cartilage ECM. More importantly, the PHBV-g-QUE fibrous scaffold significantly promotes maturation of neo-cartilage tissue and cartilage regeneration in vivo compared with PHBV fibrous scaffold. It suggests that the PHBV-g-QUE fibrous scaffold can be potentially applied in CTE for clinic cartilage defect repair.
